# Repertory of the Back

**Published:** 1899-04

**Authors:** E. H. Wilsey

**Affiliations:** Parkersburg, W. Va.


### REPERTORY OF THE BACK.

E. H. Wilsey, M. D., Parkersburg, W. Va.

Continued from January No., p. 36.

### LUMBAR REGION.

**Digging** stitches in left loins disappearing with walking, but recurring when sitting, Dulc.

**Dinner,** sticking from left loin transversely through abdomen after dinner; worse below umbilicus and toward right groin, Ran-b.

**Distress,** aching in lumbar region, intermittent, sharp, with constant distress, Lept.

**Distressing** pain in left lumbar region before chill, *Eup-pur.*

**Dorsal,** soreness of last dorsal and first lumbar vertebra, Bell.

— pains between shoulders and in lumbar region, *Con.*

— tearing in right lumbar and dorsal muscles, worse on moving parts, Brom.

**Dragging** dull pain in lumbar region, *Ham.*

— dull, heavy pain and stiffness in back, especially across lumbar region, necessitating use of arms to raise from seat, *Hydrast.*

— heavy, dull feeling in lumbar region, with stiffness and want of elasticity, Syph.

— sensation from behind forward, resembling weak labor pains, *Sabina.*

— and aching in lumbar region, worse from motion, *Pic-ac.*

— with heaviness in lumbar region and increased urine, Kali-chlor.

— when waking in morning in lumbar region, also till P. M., Myrica.

— pain in lumbar region with leucorrhœa, *Ferr.*

— across lumbar region, Carbol-ac.

— severe in ulcerated cervix, *Merc.*

— in lumbar region with dysmenorrhœa, Sabina.

— in lumbar region, cannot move, *Phos.*

LUMBAR REGION.

**Dragging** and heavy weakening pain in lumbar region, Eryngium.

**Drawing**, pressing, tired pain in lumbar region, *Mur-ac.*

— pain as if something alive, pressure and drawing at times; tearing only distinctly felt when standing, Phos-ac.

— labor-like pains, drawing down into groins, Sabina.

— on movement like a soreness or like a spasmodic drawing, Sul-ac.

— in lumbar and dorsal muscles, with heaviness and indolence, on moving in open air, Tereb.

— and tensive pinching in loins and above hips at noon when walking and standing, Ver-a.

— pain when lying quietly in lumbar region, better by moving, Colchi.

— stitches and tensive drawing pains, much increased by moving arms upward, Con.

— pain in lumbar region waking him, worse by turning, Bry.

— acute pain in lumbar region by moving body, with increased urine, Nitrum.

— in lumbar region, spine, and thighs after coition, Nit-ac.

— pressing and stiffness in lumbar region as if broken, *Carbo-an.*

— pain in loins after stool, Mag-m.

— across loins, worse at every step, *Carbo-an.*

— pain in the loins when walking, standing and lying, feeling as if broken, *Carbo-an.*

— sensation of a cord tightly drawn across lower loins drawing down and also in, *Arn.*

— rheumatic and aching from loins to coccyx, *Carbo-veg.*

— a pressive drawing pain in small of back and loins, only while resting (sitting, standing or lying down) in daytime, disappearing in walking, Amm-c.

— in right lumbar region, worse by walking, better when sitting, Æthusa.

— through lumbar region when standing, Con.

— with pain through lumbar vertebræ on standing, Cann-i.

LUMBAR REGION.

**Drawing,** a pressive drawing in lumbar region, Sep.
— in lumbar region reaching sometimes to testicles, Psorin.
— tensive pain in loins, *Puls.*
— tearing in bones in lumbar region, Rhod.
— cutting in lumbar region beneath short ribs and in forepart of left side of lower abdomen just above pubes, Rheum.
— severe pains in lumbar region, Erigeron.
— pain in right lumbar region at 9 P. M., passing to right testicle, then dragging pain in left hypochondrium, Erigeron.
— from lumbar region to pubes with lucorrhœa, *Sabina.*
— spasmodic in lumbar region compelling him to lie still, *Sil.*
— stitching in lumbar region on rising from a seat, Sul.
— in lumbar region extends along course of ureters, *Berb.*
**Dreams,** pain in loins last two days of menses, with anxious dreams, Caust.
**Dropping,** sensation of water dropping out of a bottle in lumbar region, Medor.
**Dull** pain in lumbar region, Zing., *Caulo.*, *Ham.*, Ind., Cund., *Lil-tig.*, *Lyc.*, *Hydrast.*, Diosc., Dulc., *Phos-ac.*, Zizia., Senecio, *Gels.*, *Pareira.*, Cornus., Ustil., Tereb., Cann-i, Lycop-vir.
— pains in lumbar region after emission, with despondency and irritability, Ustilago.
— pain to testicle, worse bending spine, Diosc.
— pain in lumbar region after taking cold, *Lyco.*
— pain in loins increased by movement, Zizia.
— pain in left lumbar region, Senecio.
— pain in loins going down back of legs to lower part of calves, *Phos-a.*
— dragging pain in lumbar region, *Ham.*
— aching pain in lumbar region extending to back, Ind.
— aching in lumbo-sacral region, cannot walk, muscles will not obey, *Gels.*
— stitches in both loins with sensation as if squeezing from within outward, at every inspiration while sitting bent, Dulc.

LUMBAR REGION.

**Dull** pains in lumbar region with nephritic colic, Pareira.

— pain in lumbar region with drowsiness and lassitude, Cornus.

**Eating,** pain in lumbar region after eating as from a band just above hips, Cina.

**Electric** sparks like, extending into ribs from above right ilium, Caust.

**Emission,** aching in lumbar region following seminal emission, with weakness of legs, Cobalt.

**Erect,** stiffness of muscles of lumbar region while bending over for a short time, causing great difficulty when assuming an erect posture, *Hydrast.*

**Eruption,** oval patch on lower third of surface of left loin about four and three-quarter inches in length, covered with a powder resembling chalk; the edges of a yellow color; small eruption having the appearance of millet, *Hydrocotyle.*

— itching miliary eruption on lumbar region, Arundo.

— of pimples in lumbar region, China, Clem.

**Eructation,** throbbing pain in lumbar region during shaking chill with eructation, *Nux-v.*

**Evening,** sticking in loins midway between hypochondrium and iliac crests in evening when playing on piano, Stram.

— quivering of muscles of right lumbar region in evening, Agar.

— pain in lumbar region in evening, Sumbul.

**Expiration,** stitches of bones in lumbar vertebræ on expiration, Sul.

— stitches in left lumbar region, worse by expiration, Amm.

**Exercise,** weakness of loins after exercise, Plant.

**Exertion,** pains in loins worse by slightest exertion, obliging him to sit down and rest, Cann-s.

**Fall,** cramp-like pain in lumbar region after long standing; when attempting to walk feels as if would fall, Thuja.

— a feeling as if falling to pieces in lumbar region, with a desire to bind them up tightly, Trill.

LUMBAR REGION.

**Fatigue** and heaviness of lumbar muscles, Codein.

**Fatigued** pain in loins as if he had stood a long time, *Cina*, Arn.

**Feet,** pain in lumbar region then going down limbs to feet, Sang-c.

**Fever,** severe pain in lumbar region with fever, Tereb.

**Fire,** burning heat in lumbar region as if clothes were on fire, Ars-iod.

**Flannels**, severe rheumatic pains in lumbar regions or muscles contracted by removing flannels too soon, Tereb.

**Flatus,** pain in lumbar region in morning as from incarcerated flatus low down in hypochondrium, *Nux-v.*

— pain in the loins as from flatus which could not pass, toward evening when sitting, Helleb.

**Flea-bite**, burning itching like a flea-bite in region of the left loin so that he shudders, Alum.

**Fleeting** stitches in loins, *Cocc-c.*

**Flying** pains in muscles with persistent aching in loins and occiput, worse on movement, Lyc-vir.

**Formication** in lumbar region, Arundo., Phos-ac., Canth.

— in left lumbar region, Canth.

— painful, rising to shoulders, settling in left clavicle, Arundo.

— violent pains in 1st and 2d lumbar vertebræ when turning, Agari.

— in left lumbar region sensation of coldness and formication on spot size of a hand, Canth.

**Fullness** and pressure in lumbar region, Arn.

— in lumbar region with hæmaturia, *Nux-v.*

**Gnawing** in a spot in lumbar region after pressure upon it, only a bruised pain, Sul.

— in lumbar region at night, worse in bed, Lil-tig.

— in lumbar region, Niccol.

**Grasping**, pinching in left loins on rapid walking, impeding respiration, better by pressure, Drosera.

**Griping** in lumbar region, Phyto., Sul.

### LUMBAR REGION.

**Griping** and sticking by false ribs, Stann.

**Gurgling** in right lumbar region, Sepia.

— in left side of lumbar region, extending across it, Lyco.

**Headache,** lumbago alternating with headache, Aloe.

**Heat,** burning in lumbar region as if clothes were on fire, Ars-iod.

— in whole right lumbar region, Sarracenia.

— flushes of heat in lumbar region, Calc-phos.

— dry with coldness of back, Sul.

— flushes of heat in all directions from lumbar region, Bapt.

**Heaviness** in lumbar region with dragging and increased urine, Kali-chl.

— in lumbar region increases pain in legs, *Phos-ac.*

— and fatigue in lumbar muscles, Codein.

— and indolence on moving in open air, with drawing in lumbar and dorsal muscles, Tereb.

— and pressure at end of lumbar vertebræ, Amm.

**Heavy** and constant dull pain in lumbar and sacral region, *Phyto.*

— pain and weakness in lumbar region, Lil-tig.

— pain in lumbar region, worse by stooping, Diosc.

— pulling in right lumbar region at 3 P. M., Sep.

— sensation of a heavy weight in the lumbo-dorsal region, better by lying on left side, with increase of temperature and sensibility of parts affected, *Coloc.*

— dragging dull pain and stiffness of back, especially across lumbar region, necessitating the use of arms to raise from seat, Hydrast.

**Inflammation** of right side toward lumbar region with pain and extravasation of blood, Crotal.

**Inspiration,** tensive stitch pain in the right loin felt only during inspiration and most violent when lying on back, *Coloc.*

— dull stitches in both loins with sensation as if squeezing from within outward, at every inspiration while sitting bent, Dulc.

— piercing pain in right loin during inspiration, Aur-mur.

LUMBAR REGION.

**Intense** pain in left lumbar region between floating ribs and ilium, Vib-op.

— pain in left lumbar region above hip as if he had strained part, worse by standing and especially sitting, Valer.

**Itching** burning like a flea-bite in the region of left loin so that he shudders, Alum.

— erosive itching on left side of loin inciting him to scratch, Dig.

— in lumbar region, must scratch it raw, *Bar-c.*

— erosive about the lumbar vertebræ and in other parts of the body, also on the thigh, Phos-ac.

**Jerking,** rending, cutting, shooting in lumbar region, *Calc-phos.*

— in lumbar region in evening in bed, Sul.

— stitches in right lumbar region at 7 P. M., Carboneum.

**Jerk-like** pain, like a cramp, when sitting and lying, Bry.

**Jerks,** severe, single stabs in jerks as if with a fork, close above the right hip beside the lumbar vertebræ, Dulc.

— liver sticking to back and thence to urethra, sticking jerks on every motion, Lach.

— sudden violent jerks in lumbar region during a walk in open air arresting breathing, Ran-scler.

**Knee,** numbness and lameness in right lumbar region from vertebræ to crest of ilium and inner side of thigh down to knee, Ars-met.

— sticking in the lumbar region extending to knee in morning, Psorin.

**Knife,** pain as from a knife sticking through loins, cannot walk, *Kali-b.*

**Labor-like** pain in lumbar region, *Coff.*, *Vib.*, *Carbo-v.*, Eupat-pur., *Kali-c.*, *Kreos.*, *Lyco.*, *Pulsat.*, Sec., Sabin., Fer., *Sep.*, *Aloe.*, *Coccul.*, *Lil-tig.*, *Cycl.*

— pain from lumbar region into buttocks and thighs with impending abortion, *Kali c.*

— pains in lumbar region with dysmenorrhœa, pains down each side of abdomen to pubes, *Cycla.*

LUMBAR REGION.

**Labor**, pains running through to front at intervals of a few minutes.
occasionally shooting down to glutei muscles, *Kali-c.*
— pains worse standing, with prolapsus uteri, *Aloe.*
— pains with hour-glass contraction of uterus, *Coccul.*
— pain goes to loins and across to pubic bone, *Vib.*
**Labor**, dragging sensation from behind forward resembling weak labŏr pains, *Sabin.*
**Lame** feeling in lumbar region in morning without pain, Selenium.
**Lameness**, pain in sacro-lumbar region with lameness and stiffness, Helon.
— and numbness in right lumbar region, from vertebræ to crest of ilium and inner side of thigh down to knee, Ars-met.
— in lumbar region, *Cup-ars.*, *Æscul-hip.*
— in lumbar region, painful and weak, Lach.
— in lumbar region, with lucorrhœa, *Caustic.*
**Lame**, sore feeling in lumbar region, Onos., *Lept.*
— stiff feeling in back and legs, Cap.
— in loins after taking cold, *Dulc.*
— feeling in lumbar region after difficult parturition, *Nux-v.*
— feeling follows emissions with lascivious dreams awakening him, Selen.
— feeling with prolapsus uteri, Æscul-hip.
**Lancinating**, deep pains around loins, Ginseng.
— sharp pain in lumbar region, Senecio.
— fine tearing on right side beside the lumbar vertebræ, passing away every time he presses upon it, Aur-fol.
— in lumbar region, Cup-m., Lac-can., Asaf.
**Languor**, tired aching in lumbar region, with a feeling of languor, Zinc.
— tired aching over the crest of the ilium and down into pelvis, right side, with general languor and desire to lie down, later the tired aching, worse morning, Spig.
**Legs**, aching in loins shooting down legs, *Sil.*

LUMBAR REGION.

**Legs,** severe pains in lumbar and hypogastric region, extending down legs, *Ham.*

**Lie,** pain in lumbar region at night so must lie on abdomen, Nit-ac.

**Lifting,** sudden stitches in lumbar region when lifting, he had to walk bent forward and had stitches if his foot struck against anything, Sepia.

**Limbs,** numb feeling extending from lumbar region to lower limbs, *Acon.*

**Lumbago,** *Bry.*, *Sul.*, *Rhus-t.*, *Nux-v.*, *Arn.*, *Actea-r.*, *Berb.*, *Coloc.*, *Dulc.*, *Lyco.*, *Fer-phos.*, *Nux-m.*, *Nat-m.*, *Magn-m.*, *Phyto.*, *Puls.*, *Secale*, Rhus-v., Guaicum, *Kali-c.*, Calc-fl., Val., Med., Ant-t., Cal-phos., Colch., Ferr., *Kali-s.*, Led., *Nat-s.*, Oxal-ac., Pet., Rhod., Ruta., Staph., Tereb., Ars-m., Arundo, Asclep-tub., Chromic-ac.

— alternating with headache, Aloe.

— from lifting: or myalgia of the great muscles of the back, *Sang.*

— pain worse after, not during motion, *Rhus-t.*

— and rheumatism of the back, *Nux-v.*

— chronic for several years' standing, with difficulty in walking, Oleum-jac.

— all night, goes off on rising, Ferr.

— pain in loins like lumbago, Asclep-tub.

— caused by straining and lifting, Med., Sang.

— crampy when standing in street without overcoat, better by external support and adjusting position, Chin-s.

— if Bry. has not relieved sufficiently and pain, worse from slightest motion, *Lyco.*

— pain extends from back around body, down leg, with red and mucous sediment in urine, Berb.

— from strains; pains worse after rest, better after moving a little and from warmth (when Rhus-t. did no good), *Calc-fl.*

— and crick in back, Calc-phos.

— back is very stiff every morning, *Phyto.*

LUMBAR REGION.

**Lumbago,** and sciatica, the vertebræ feel as if rubbed against each other, *Ant-t.*

— must move, yet motion gives no relief, *Puls.*

— can hardly walk, *Sul.*

— from a cold, *Rhus-v.*

— must lie on left side at night, *Sul.*

— by motion, disturbs sleep at night, *Sul.*

**Lung,** sticking in lumbar region, extending to left lung, Sepia.

**Lump,** sensation of a lump the size of a hen's egg, rising and falling in right lumbar region, *Hydrast.*

**Lying,** pain when sitting as if back had been bent too long, and as if one had been lying on it too long, Rhod.

— tensive stitch pain in the right loin; felt only during inspiration and most violent when lying on back, *Coloc.*

— bruised feeling in region of loins, especially while lying and sitting, Agari.

— pain in lumbar region, worse sitting and by lying, Tabac.

— drawing pain in loins when walking, standing, and lying, feeling as if broken, *Carbo-an.*

— on back with great weariness, going to feet from lumbar region, *Lyco.*

**Menses**, bruised pains in loins during menses, Bary-c.

— pain in loin the last two days before menses, with anxious dreams, Caustic.

— pain in lumbar region during menses, worse at night, Nat-c.

— pain in lumbar region during menses, Ratan.

— pain in loins as before menses when sitting, Carbo-a.

— aching before menses in lumbar region, *Magn.*

— aching across lumbar region during menses, *Lach.*

— aching across lumbar region with menorrhagia, *Coccus.*

— bruised pain during menses, Baryta-c.

— scanty flow, with colicy pain, Sul.

— cutting in lumbar region before menses when too early, *Ol-an.*

— cutting during menses, *Arg-n.*

— drawing in lumbar region before menses, *Hyos.*

LUMBAR REGION.

**Menses,** labor-like pain in lumbar region during menses, *Puls.*
— pain in lumbar region before menses, with debility, pressure in stomach, water brash, and pain in liver, thick and black flow, *Nux-m.*
— pain before, in metrorrhagia, *Sepia.*
— pain with early menses, Niccol.
— pain with too early, profuse and long duration, Ratanhia.
— tearing during menses, *Caust.*
— pain at appearance of menses, scarcely permits breathing, *Asar.*
— pain downward during, *Nux-m.*
— pain in lumbar region during menses, *Amm-c.*, *Kali-c.*, *Caust.*, Brom., Berb., *Magn-c.*, *Tarant.*, *Kalmia.*, Lach., Lact-ac.
— pain during first day of menses, *Lach.*
— pain in lumbar region when menses should appear, *Spong.*, *Ham.*
— as if menses were coming, worse early in the evening and in a close room, better in open air and moving about, Vib.
— pain in lumbar region after menses, Berb.
— pain in lumbar region with dysmenorrhœa, *Xan.*, *Lach.*
— pressing in lumbar region during menses, *Carbo-an.*, *Lach.*
— pressing downward like a stone during menses, *Puls.*
— throbbing in lumbar region before menses, *Nit-ac.*
— stitches into uterus with menses, *Nat-m.*
— pain like a weight during, *Kali-c.*

**Morning,** pain in lumbar region in morning with general indolence, Ran-b.
— pain on rising in loins, under scapula and in pelvis, Cedron.
— pain in lumbar region, most painful at 9 A. M., Chromic-ac.
— acute pain in loin in the morning, Stann.
— pressure in left side near lumbar region, worse from motion of body and pressure, *Nat-s.*

**Motion,** dull aching pain, worse by motion, in lumbar region, Æscul.
— backache in cervical, lumbar, and sacral region, worse by motion, Æscul.

LUMBAR REGION.

**Motion**, lumbago pain, worse after and not during motion, *Rhus*.

**Moving**, pain in lumbar vertebræ when moving, Ars-sul-fl.

**Myalgia**, of the great muscles of the back, *Sang-c*.

**Neck**, pain in nape of neck follows lumbar pains, Sil.

**Night**, pain in lumbar region during menses, relieved at night, Nat-c.

— pain in lumbar region, withered sensation, worse at night and from motion, Podo.

— lumbago all night, goes off on rising, *Ferr*.

— severe pains in the lumbo-sacral region, mostly during latter part of night, *Carbol-ac*.

**Noon**, sticking in loins at noon, Hyperi.

**Numb** feeling extending from lumbar region to lower limbs, *Acon*.

— stitching pain in small of back so severe upon motion that he gets upon arms and knees in order to obtain relief, pain unendurable in any other position, *Colocy*.

— as if dead in lumbar region after suppressed foot sweat, *Sil*.

**Numbness** in lumbar region, Spong.

— in lumbar muscles with lumbago, Gnaphal.

— worse by external hard pressure, *Berb*.

— and lameness in right lumbar region from vertebræ to crest of ilium and inner side of thigh down to knee, Ars-m.

**Pain** in loins, Ver-alb., Vipera, Sul., *Curare*.

— and weakness in lower lumbar region, *Amyl-nit*.

— in both loins as if surrounded by bands, Caustic.

— across lumbar region, suffers much from it, *Sepia*, *Arg-n.*, Cund.

— across lumbar region or vertebræ, Gambog.

— between shoulders and in lumbar region, *Con*.

— as after a blow had been received above the hip close by the lumbar region or vertebræ, Dulc.

— dull in loins going down back of legs to lower part of calves, *Phos-a*.

LUMBAR REGION.

**Pain** as from dislocation in loins when sitting and turning the body in walking, *Hepar.*

— in lumbar region when lying quietly a drawing, better by moving, Colchi.

— in lumbar region after eating as from a band above hips, Cina.

— in lumbar region in evening, Sumbul.

— in loins on slightest exertion, obliging him to sit down and rest, Cann-s.

— in lumbar region, then going down limbs to feet, Sang-c.

— severe in lumbar region with fever, Tereb.

— in left loin, Sumbul.

— in loins last two days before menses with anxious dreams, Caustic.

— in morning, on rising, in loins, under scapula and in pelvis, Cedron.

— in lumbar region most painful in morning, 9 A. M., Chromic-ac.

— in lumbar vertebræ when moving, Ars-s-fl.

— in nape of neck follows lumbar pain, *Sil.*

— in lumbar region of spine followed by apoplexy and paralysis, *Bary-c.*

— in loins, as if all power was going and the back was turning to pulp, Colch.

— pulsating in lumbar region, Cimic.

— as though the lower lumbar vertebræ would separate when bending forward, *Chelid.*

— severe in lumbar and sacral region, *Amm-mur.*

— in loins like toothache when she *stands*, which makes her feel sick and good for nothing, *Colchi.*

— as if squeezed in lumbar region, Caustic.

— in loins seems to arise in a spot which is tender to pressure, about an inch to right of middle lumbar spine, Chromic-ac.

— in lumbar region at 5 P. M., extending to the testes, Abrot.

— in lumbar portion of spine; myalgic; with induration of the testes, *Med.*

### LUMBAR REGION.

**Pain** in right lumbar region and in anterior portion of right thigh, Cup-ars.

— in loins, with vomiting, Ars-h.

— in loins when walking on uneven ground or from a misstep, Podo.

— in lumbar region on rising from a seat and on beginning to walk, better by walking, Tabacum.

— in region of lumbar vertebræ, *Arn.*

— in lumbar region, Ars-h., *Sul-ac.*, *Doryphora.*, Eryngium (at 2 P. M.).

— in the back and loins, Chromic-ac.

— in lumbar region of spine, Zinc.

**Paralysis,** muscular paralysis of lumbar region, also of lower extremities, from cold, *Coccul.*

— a sensation of paralysis along lumbar vertebræ immediately above the border of the os ilium ; this hinders him in walking when he has risen, Agari.

**Paralytic** condition of lumbar and sacral muscles, *Gels.*

— sensation from weakness back in the loins, worse by walking or standing, Agari.

— pain in loins at 10 P. M., in bed, with dullness and pain in head, Kalmia.

— feeling in lumbar region, *Berb.*, Euphorb.

— feeling at junction of lumbo-sacral vertebræ, *Phos.*

— feeling, painful, would like to stretch, better bending backward, Sabina.

**Piercing** sharp pain in right loin, merely on inspiration, Aur-fol.

**Pierced** pain in loins, as if pierced by red-hot irons, two mornings, worse by movements, arresting the breath, and nearly causing faintness ; next day pain, as if strained, preventing him from straightening himself or stooping, Bufo.

**Pinching,** tensive and drawing in loins and above hips at noon when walking and standing, Ver-alb.

— and grasping in left loin on rapid walking, impeding respiration, by pressure, Droser.

### LUMBAR REGION.

**Pinching,** crampy pain in lumbar region and buttocks, *Caustic.*

**Pressing** pain in articulation of lumbar vertebræ and sacrum, left side; pressing on spot causes pain down thigh to knee, Nat-phos.

— drawing and stiffness in lumbar region, as if broken, *Carbo-an.*

**Pressive,** shooting pain on the external right side lumbar vertebræ, worse in morning, Mez.

**Pressive** drawing in left lumbar region, Sepia.

— drawing pains in small of the back and loins only while resting in daytime, disappearing when walking, Amm-c.

— violent pain in lumbar region; worse when at rest, so that she has to walk about to alleviate it; by coughing it is so much worse that she must scream, Phos.

**Pressure** above the loins, with sensation in legs as if they were stiff and bandaged, Nat-m.

— in lumbar region, Spongia.

— muscles tender on pressure and stiff, especially on first moving after repose, *Cactus.*

— downward, above left hip, Stann.

— and heaviness at end of lumbar vertebræ, Amm.

— lower lumbar region is slightly sensitive to pressure, *Arg-n.*

— and fullness in lumbar region, Arn.

— outward in left loin, Taraxac.

— and tearing in lumbar region, Carboneum.

— pain in loins seem to arise in a spot which is tender to pressure about an inch to right of middle lumbar spine, Chromic-a.

— in left side near lumbar region; worse from motion of body and pressure, *Nat-s.*

— on left side near lumbar vertebræ and upon upper border of os innominatum, Aur-fol.

**Pricking** in loins, Aur-mur.

**Psoas** abscess, Symphytum.

— first left, then right; latter discharged more than a quart of offensive greenish pus when opened, Syphil.

LUMBAR REGION.

**Psoas** muscle inflamed, *Asaf.*, Bryonia., Cal-carb.
**Pulling** in lumbar region, *Hyos.*
— heavy in right lumbar region at 3 P. M., Sepia.
**Pulsating** stitches in lumbar region, Sul.
— pains in lumbar region, Cimicif.
**Pulsation** in lumbar region, Nit-ac.
**Quivering** of muscles of right lumbar region in evening, Agari.
**Respiration**, above the right loin a pain that obstructs, Carbo-veg.
— grasping, pinching in left loin on rapid walking, impeding respiration, better by pressure, Drosera.
— tearing in lumbar muscles, impeding respiration, *Kali-c.*
**Retching**, violent pains in lumbar region and sacrum; slightest effort to move causes retching and cold sweat, *Ant-t.*
**Rheumatic** pains in lumbar region, *Berb.*, *Bryon.*, *Ant-c.*, *Sul.*, Eup-pur., Phyto., Stram. (Ustil., worse by walking), Cact., Iod.

